# Semiology, clustering, periodicity and natural history of seizures in an experimental occipital cortical epilepsy model

**DOI:** 10.1242/dmm.036194

**Published:** 2018-12-14

**Authors:** Bao-Luen Chang, Marco Leite, Albert Snowball, Andreas Lieb, Elodie Chabrol, Matthew C. Walker, Dimitri M. Kullmann, Stephanie Schorge, Robert C. Wykes

**Affiliations:** 1 Department of Clinical and Experimental Epilepsy, UCL Institute of Neurology, Queen Square, London WC1N 3BG, UK; 2 Section of Epilepsy, Department of Neurology, Chang Gung Memorial Hospital at Linkou Medical Center and Chang Gung University College of Medicine, Taoyuan, Taiwan

**Keywords:** Tetanus toxin, Occipital cortical epilepsy, Circadian rhythm, Seizure clustering, Periodic pattern, Prediction

## Abstract

Focal neocortical epilepsy is a common form of epilepsy and there is a need to develop animal models that allow the evaluation of novel therapeutic strategies to treat this type of epilepsy. Tetanus toxin (TeNT) injection into the rat visual cortex induces focal neocortical epilepsy without preceding status epilepticus. The latency to first seizure ranged from 3 to 7 days. Seizure duration was bimodal, with both short (approximately 30 s) and long-lasting (>100 s) seizures occurring in the same animals. Seizures were accompanied by non-motor features such as behavioural arrest, or motor seizures with or without evolution to generalized tonic-clonic seizures. Seizures were more common during the sleep phase of a light-dark cycle. Seizure occurrence was not random, and tended to cluster with significantly higher probability of recurrence within 24 h of a previous seizure. Across animals, the number of seizures in the first week could be used to predict the number of seizures in the following 3 weeks. The TeNT model of occipital cortical epilepsy is a model of acquired focal neocortical epilepsy that is well-suited for preclinical evaluation of novel anti-epileptic strategies. We provide here a detailed analysis of the epilepsy phenotypes, seizure activity, electrographic features and the semiology. In addition, we provide a predictive framework that can be used to reduce variation and consequently animal use in preclinical studies of potential treatments.

## Abstract (precis)

**Summary:** Tetanus-toxin-induced occipital cortical epilepsy is a rodent model of acquired focal neocortical epilepsy well-suited for preclinical evaluation of novel anti-epileptic strategies.

## INTRODUCTION

Animal models of epilepsies are an invaluable tool to test novel anti-epileptic treatments. At present, the most commonly used rodent models of epilepsy rely on chemoconvulsants such as kainic acid or pilocarpine, or on electrical stimulation, to induce status epilepticus (SE), which is followed by spontaneous recurrent seizures. These approaches predominantly model mesial temporal lobe epilepsy (mTLE) (Kandratavicius et al., 2014). Various mouse and rat genetic models have also been used, mainly to model primary generalized epilepsy. *In utero* electroporation can be used to manipulate phosphatase and tensin homolog (Pten) or related signalling molecules to generate malformations of cortical development (Wong and Roper, 2016). Although all these models are useful, there is a need for a model of adult-onset neocortical epilepsy with a well-defined focus. Focal epilepsy has been estimated to account for ∼60% of human cases (Téllez-Zenteno and Hernandez-Ronquillo, 2012). Many new antiepileptic drugs and surgical interventions have been introduced for epilepsy treatment; however, approximately 20–30% of epilepsy patients continue to experience uncontrolled seizures (Kwan and Brodie, 2000; Loscher et al., 2008). Surgical resection is only appropriate in the minority of cases where the epileptogenic zone is not in the eloquent cortex and therefore focal epilepsy treatment represents a major unmet need. This study characterises an animal model of adult-onset focal neocortical epilepsy that can support studies of potential treatments.

Tetanus neurotoxin (TeNT) is a metalloprotease (zinc-dependent protease) that specifically targets neurons (Schiavo et al., 1992b). TeNT consists of a heavy chain and a light chain with a disulphide bond linked between both chains. Intact TeNT is necessary for uptake by synaptic terminals since the function of the heavy chain is for specific binding and internalization into neurons, as well as retrograde axonal transport (Schiavo et al., 2000). The light chain is proteolytic, and it is inactive when bound to the heavy chain. The light chain can be activated by cleavage of the inter-chain disulphide bond once TeNT is inside cells (Schiavo et al., 2000). The activated light chain specifically cleaves synaptobrevin, which is a vesicle-associated membrane protein (VAMP) and a component of the SNARE (SNAP REceptor) complex that is critically responsible for presynaptic neurotransmitter release (Schiavo et al., 1992a; Montecucco and Schiavo, 1993). As a result, TeNT causes vesicles to fail to fuse with the presynaptic membrane, disrupting exocytosis and the release of neurotransmitters. Furthermore, TeNT predominantly interferes with presynaptic vesicle release of inhibitory neurotransmitters, both γ-aminobutyric acid (GABA) and glycine, in the central nervous system, and therefore disinhibits the local circuitry (Albus and Habermann, 1983).

Injection of TeNT into the hippocampus has been used to produce a rat model of mTLE (Jefferys et al., 1995; Jiruska et al., 2010). In addition, a rat model that may share similarities with human epilepsia partialis continua (EPC) has been induced by TeNT injection to the motor cortex (Nilsen et al., 2005). However, the major feature of this model is brief bursts of high frequency (<1 s, 120–160 Hz) electroencephalogram (EEG) activity. Although discrete long-lasting epileptic discharges and generalized motor convulsions can be elicited with higher doses of TeNT, this is relatively poorly tolerated, with a high proportion of animals experiencing weight loss or death following a severe seizure (Nilsen et al., 2005; Wykes et al., 2012). A mouse model using TeNT injected into the visual cortex has been reported, although it mainly elicited electrographic epileptiform discharges with clusters of high-amplitude spikes lasting for ∼4 s; behavioural correlates were not reported (Mainardi et al., 2012).

The goal of this study is to characterize a rat occipital cortex TeNT model to allow studies of experimental treatments of focal neocortical epilepsy. We comprehensively characterize the epilepsy phenotypes, seizure activity, electrographic features and the semiology. We identified a diurnal fluctuation in seizure frequency, as well as non-random clustering and periodic phenomena. Finally, we further provide a prediction profile on seizure activity in this TeNT model of occipital cortical epilepsy, which can reduce the number of animals required to power studies using this model.

## RESULTS

### Tetanus toxin injection into visual cortex produces acquired occipital cortical epilepsy in rats

**Fig. 1.**
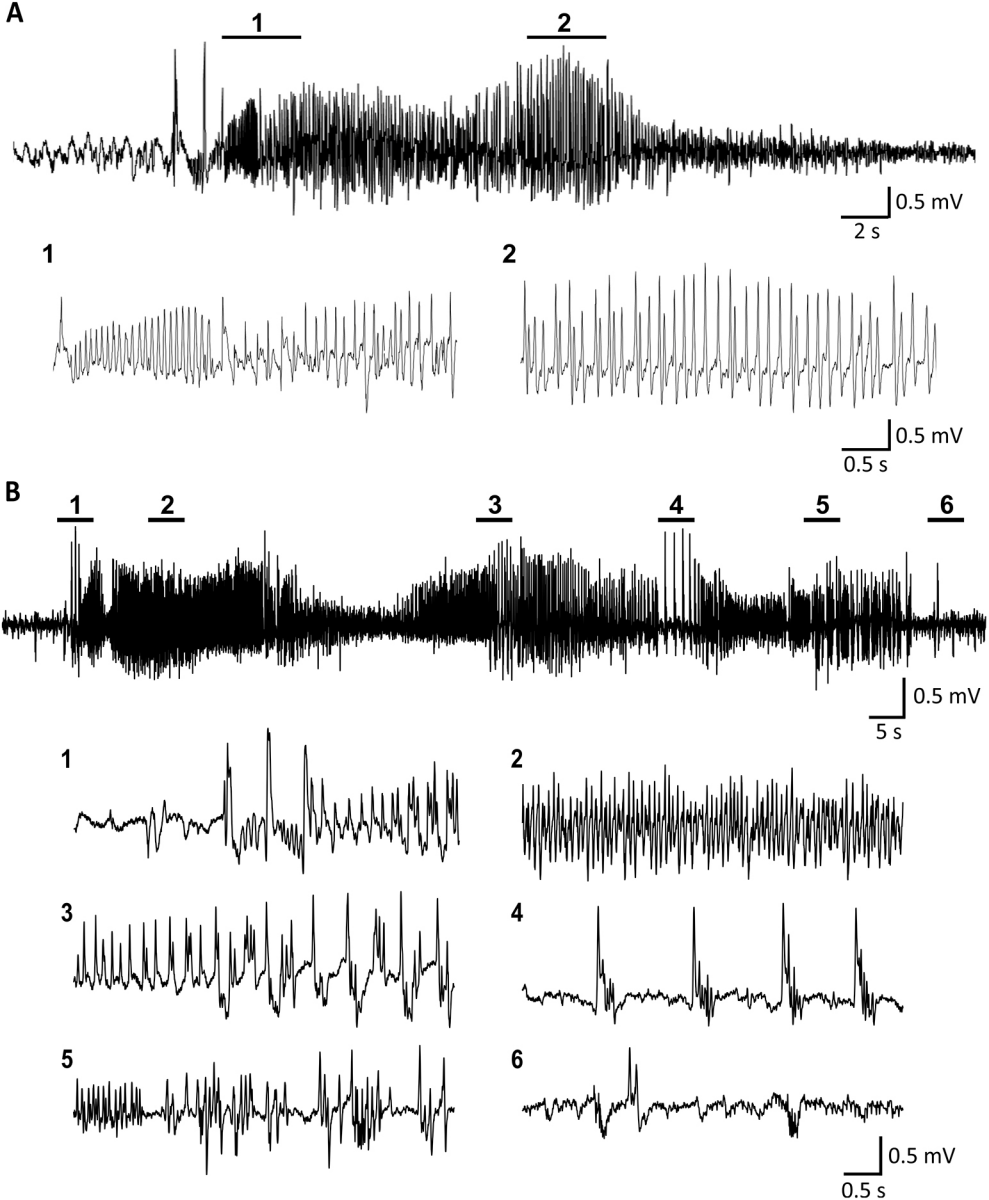
**Representative ECoG features of ictal discharges from Sprague Dawley rats.** (A) A short seizure lasting <40 s and (B) a long-lasting seizure (>100 s). The seizures start with fast activity evolving to high-amplitude and low-frequency spikes.

We established a rat model of occipital cortical epilepsy by administration of TeNT into the primary visual cortex of three different rat strains, which produced clear long-lasting spontaneous seizures in all strains tested (Fig. 1 and Fig. S1). The electrocorticogram (ECoG) at seizure onset was often characterized by low-amplitude fast activity, which evolved in amplitude and frequency. This was followed by repetitive spikes, sharp waves, polyspikes or polyspikes-and-waves, which then slowed in frequency before ECoG suppression at the end of a seizure (Fig. 1A,B). The overall success rate of TeNT-elicited epilepsy in the occipital cortex was approximately 84%. We believe that the failure to induce epilepsy in the remaining 16% of animals was due to administration of TeNT with reduced potency rather than biological resistance to the toxin. TeNT treatment and seizures were well tolerated by most animals. Neither mortality because of seizures themselves nor death due to the effects of TeNT were observed. The 16% of animals (two Sprague Dawley rats and one Lister Hooded rat in a total of 19 rats receiving TeNT injection) that received TeNT injections but did not develop seizures exhibited only epileptiform spikes. No seizures, epileptiform activity or behavioural abnormalities were observed in the vehicle control animals. As the model produced similar activity in all three strains, in order to increase the ability to compare with extensive work in models of epilepsy using Sprague Dawley (SD) rats, all the further detailed analyses were performed using SD rats only (*n*=10).

**Fig. 2.**
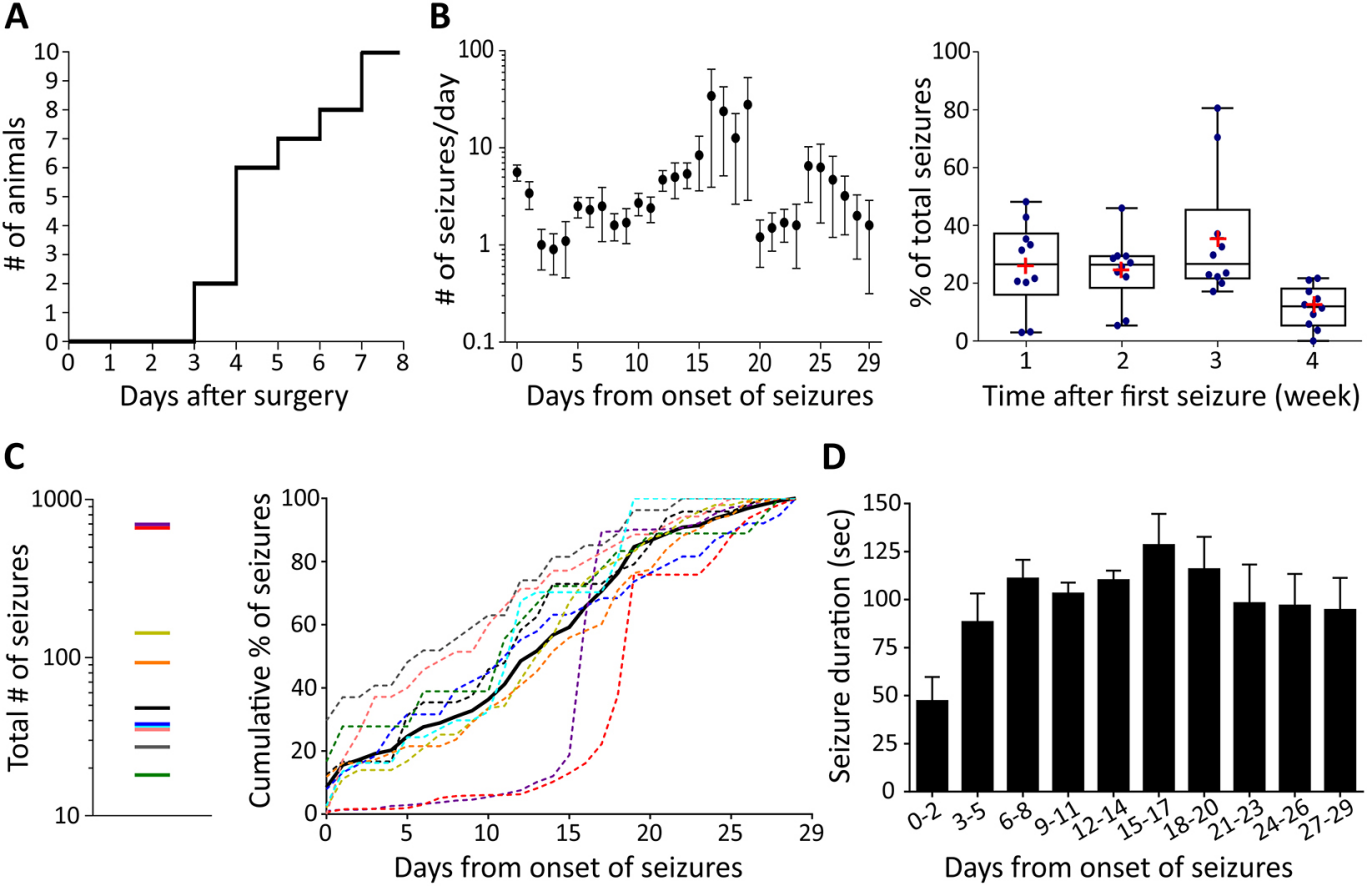
**Characterization of the TeNT model of occipital cortical epilepsy in rats.** (A) The onset of seizures after surgery was between 3 and 7 days, with most starting around 4 days post-injection of TeNT. (B) Left: daily seizure frequency (mean±s.e.m.) during the whole recording period from the onset of first seizure (time point 0). Right: the box-and-whisker graph shows the mean (red cross) and the median of % of weekly seizure frequency. (C) Total number of seizures (left panel) and the corresponding cumulative % distribution for individual animals (coloured dotted lines; right panel). The black line represents the mean of the values. (D) The average of median of seizure duration from the onset of first seizure (time point 0). Data are presented as mean±s.e.m. (*n*=10 Sprague Dawley rats).

The latent period, defined as the delay before the emergence of the first electrographic seizure after TeNT injection, ranged from 3 to 7 days (Fig. 2A). The overall trend was for seizure frequency to gradually increase over time, reaching a plateau 16–19 days after onset of seizures and decreasing thereafter in most animals (Fig. 2B, left panel). As the total number of seizures experienced among the individual animals was highly variable, ranging from tens to hundreds of seizures (Fig. 2C, left panel), we normalised to the % of total seizures during different time periods in each individual animal to compare frequency patterns between animals. The average proportion of seizures was stable in the first 2 weeks (26% in first week; 24% in second week), and increased to a peak in the 3rd week (36%) and then dropped to the lowest proportion (12%) in the last week of recordings (Fig. 2B, right panel). The cumulative distribution of seizures in most of the animals followed a similar pattern, showing a steady increase in the cumulative seizure number from the onset of seizures to 20 days after seizure onset followed by a plateau in the final days of recordings. Two animals had seizure activity that dramatically increased 15–20 days after the onset of first seizure (Fig. 2C, right panel). In all animals, the seizure duration evolved over time as epileptic activity became established. The seizure duration was relatively short in the first few days after the onset of seizures (<50 s), but gradually increased to a stable duration of approximately 100–120 s from 1 week after the onset of seizures (Fig. 2D).

### Epilepsy induced by TeNT in visual cortex presents as focal seizures with or without secondary generalization

**Fig. 3.**
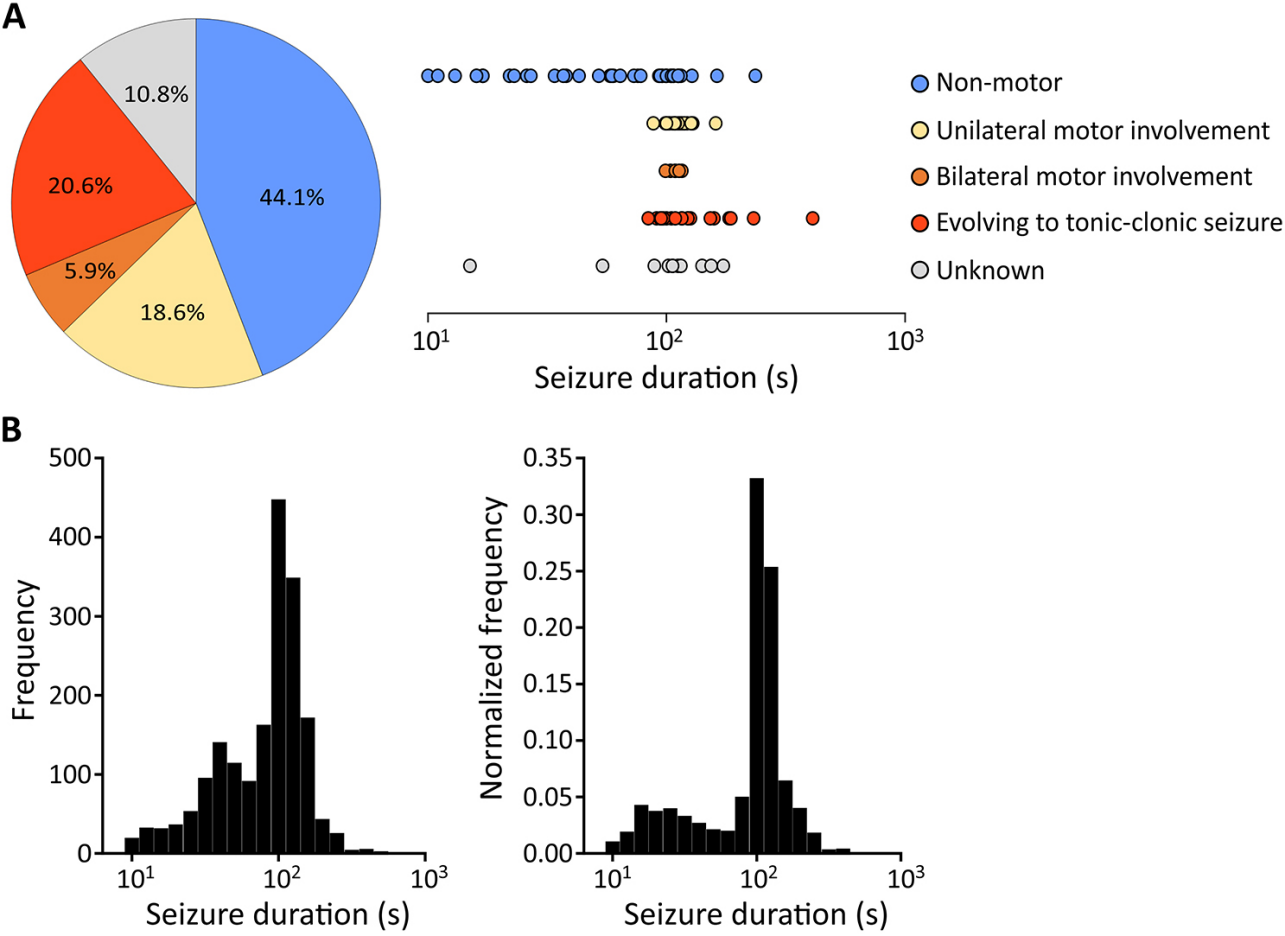
**Behavioural manifestations of seizures.** (A) Semiology classification of seizures and the distribution of seizure duration (*n*=102 seizures from eight animals). ‘Unknown’ indicates periods when the behaviours were not visible because the animals were beneath the environmental enrichment material or video signals were interrupted. All rats showed multiple types of seizures from non-motor focal seizures to focal onset with or without secondary generalisation. (B) Histogram of seizure duration from ten animals over the whole recording period. Right: the frequency was normalised to the total seizures of individual animals.

A total of 102 randomly selected seizures (out of a total of 1717 seizures observed) from eight epileptic rats with continuous 24 h/7 day video-ECoG recordings for 5–6 weeks were used to assess the correlated seizure behaviour by a neurologist (Fig. 3A). Of these, 45 seizures (44.1%) were non-motor focal seizures presenting as behavioural changes (Movie 1), including repetitive eye blinking, sudden freezing or jumping, or agitated and aggressive searching behaviour, which may be consistent with visual hallucinations arising from the epileptogenic focus. Nineteen seizures (18.6%) were classified as unilateral motor involvement, which manifested as contralateral limb twitching. Seizures propagating to bilateral motor symptoms were observed in a further six (5.9%) instances. Twenty-one seizures (20.6%) evolved to generalized tonic-clonic seizures (Movie 2). Apart from these observable seizures, associated behaviours were unobservable in 11 seizures (10.8%) due to the animals staying out of sight within the environmental enrichment material or due to transient interruption of video recordings. For non-motor seizures, the seizure duration ranged from very brief (10 s) to long (>100 s) seizures, whereas a relatively long and more consistent duration (∼100 s) was typical for seizures with unilateral or bilateral motor involvements (Fig. 3A, right panel). Seizures that evolved to tonic-clonic activity were typically long, lasting from 200 s to 400 s.

The density distribution of seizure durations for all seizures from ten animals are summarised in Fig. 3B (left panel). As the total number of seizures in two of the animals was extremely high relative to the remaining eight animals, the density distribution was corrected by normalising to total seizure counts for individual animals to avoid the two animals with highest frequency of seizures disproportionately affecting the distribution of seizure durations (Fig. 3B, right panel). A bimodal pattern was shown, where the highest probability of seizure duration was at 100–125 s and a smaller peak was at approximately 15–25 s.

### No abnormal response to photic stimulation

As Sprague Dawley rats are thought to have poor vision (Prusky et al., 2002), and this may influence detection and provocation of seizures by visual stimulation, ability to trigger seizures in this model using intermittent photic stimulation was tested in TeNT-treated Lister Hooded epileptic rats (*n*=3). However, none of the animals manifested photic-induced seizures, photoconvulsive (photoepileptiform) responses or displayed prominent ECoG changes during photic stimulation.

### Seizures occurrence during light-dark cycles

**Fig. 4.**
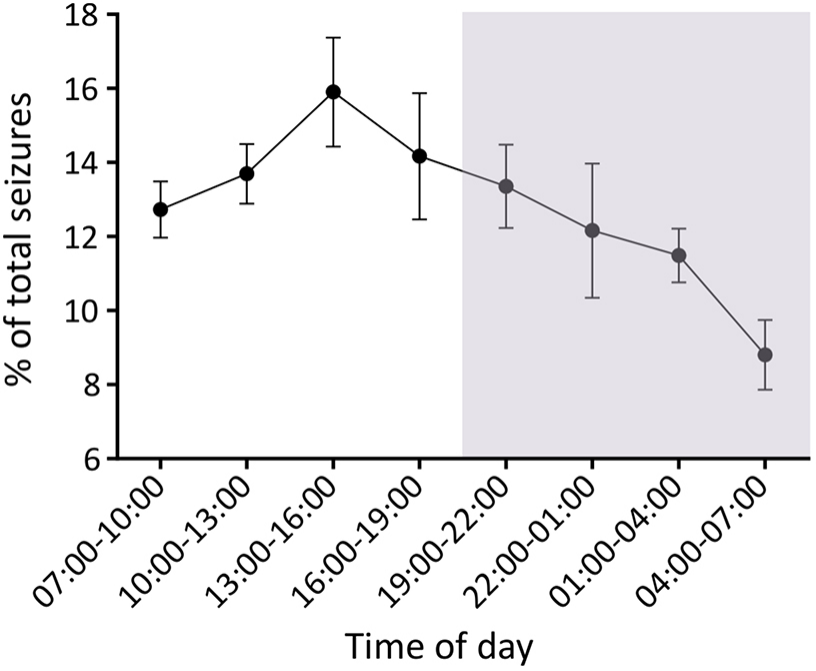
**Seizure activity during the light/dark cycles.** The grey shading represents time of light-off/activity period (7 p.m.–7 a.m.). Data are presented as mean±s.e.m. (*n*=10 animals).

To determine whether there is a correlation between circadian rhythm and seizure occurrence, the distribution of seizures during day (light-on) and night (light-off) periods was analysed. The proportion of seizures occurring during different time periods was normalised within individual animals to control for the variability of seizure frequency between animals. For seven of ten animals, seizures predominantly occurred during the light periods (sleeping periods); one animal had equal seizure frequency during light and dark; and two animals had slightly higher seizure counts during the dark periods (active periods; Fig. S2). Overall seizure frequency gradually increased from the beginning of sleeping phase (7 a.m.), reaching the highest seizure activity during the mid-stage of sleep (1 p.m.–4 p.m.), and slowly subsiding during the active dark period (7 p.m.–7 a.m.) (Fig. 4). There was no significant difference of seizure duration between day and night (Wilcoxon matched-pairs signed rank test, *P*=0.275).

### Clustering and periodicity of seizures

**Fig. 5.**
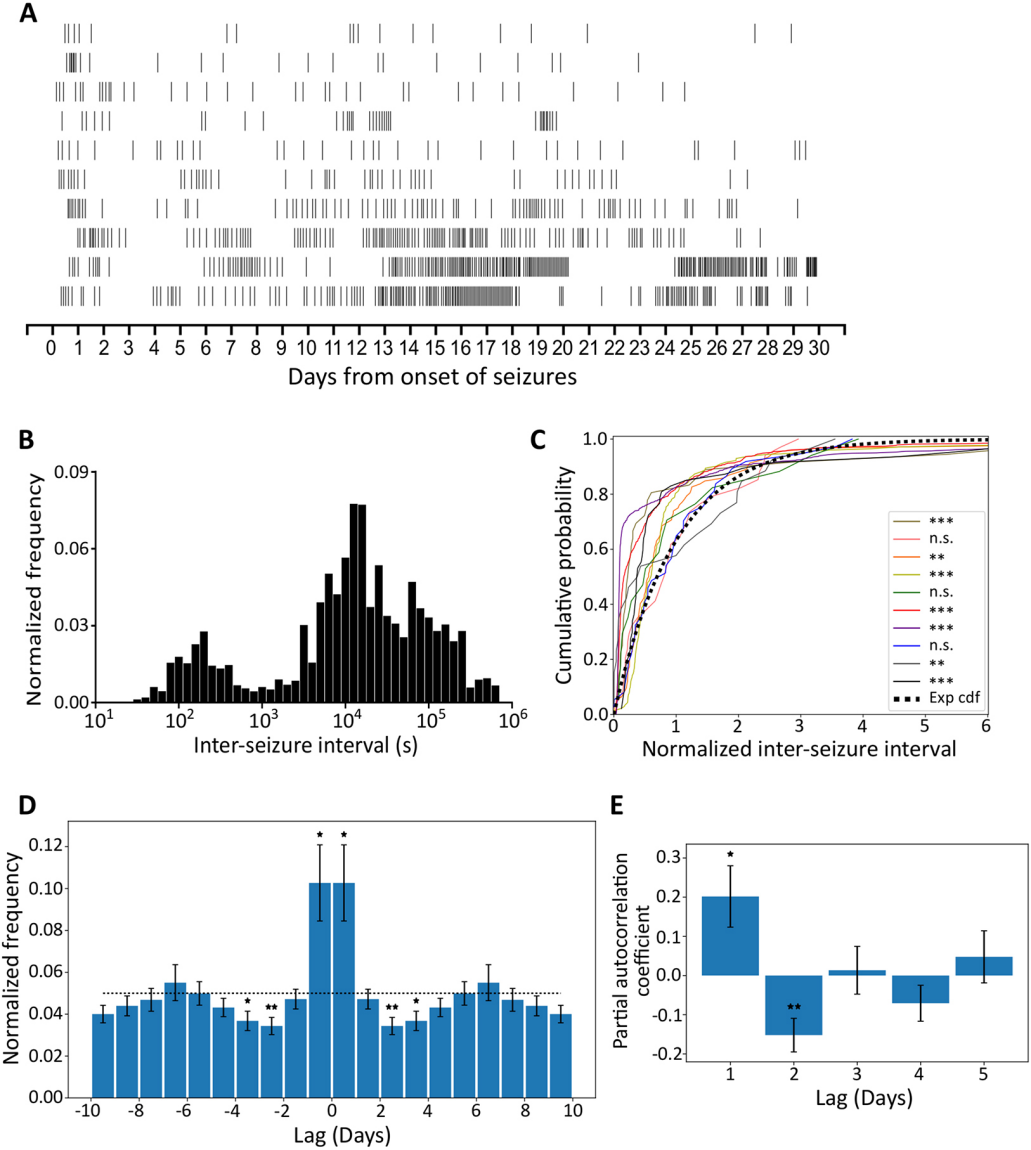
**Seizures tend to cluster.** (A) Raster plots of all seizures from onset of seizures over the whole recording period. (Each row represents the seizure distribution for an individual animal.) (B) Histogram of ISI from ten animals. The frequency was normalised to the total seizures of individual animals. (C) Deviation between individual animals (colour-coded) normalised ISI distributions and the exponential cumulative distribution function (Exp cdf). (Lilliefors-Kolmogorov–Smirnov test, **P<0.01, ***P<0.001, n.s.=not significant.) (D) Autocorrelation function. Data are re-calibrated for a short acquisition period (see Materials and Methods) and presented as mean±s.e.m. The dotted line represents the expected value of a uniform distribution. First-, third- and fourth-day counts differ significantly from the uniform distribution (two-tailed one-sample *t*-test, *P<0.05, **P<0.01). (E) Partial autocorrelation coefficients for daily seizure counts (mean± s.e.m.). The first and second coefficients differ significantly from zero (two-tailed one-sample *t*-test, *P<0.05, **P<0.01).

The distribution of seizures for individual animals over the recording period was not homogeneous (Fig. 5A). The inter-seizure intervals (ISIs) were bimodally distributed with most occurring between 3 and 4.5 h (Fig. 5B). However, no obvious correlation between seizure duration and ISIs was found (Fig. S3). To determine whether the temporal distribution seen was a consequence of the dissimilar seizure rates between different animals, the ISI distributions for individual animals were compared to exponential distributions (Fig. 5C). Indeed, most ISI distributions (7/10) differed significantly from exponential distributions, implying that seizure occurrence does not follow a homogeneous Poisson process, and is not random.

To characterise the temporal structure of seizure occurrence, we made a normalised peri-seizure histogram; this demonstrated that, on average, seizures tended to cluster (Fig. 5D), i.e. there is a significantly higher probability of seizure occurrence if a seizure has occurred in the previous 24 h (*P*=0.02). This probability decreases to values significantly below the expected value for the period between 48 and 96 h after seizure occurrence (*P*=0.006 and *P*=0.02). This distribution indicates that, after a cluster of seizures, there is a refractory period where seizures are less likely to occur. Partial autocorrelation analyses on daily seizure occurrence indicates that the seizures follow a periodic pattern (positive first coefficient, *P*=0.03, and negative second coefficient, *P*=0.008) (Fig. 5E).

### An approach to refine the model by predicting the number of seizures from early data

**Fig. 6.**
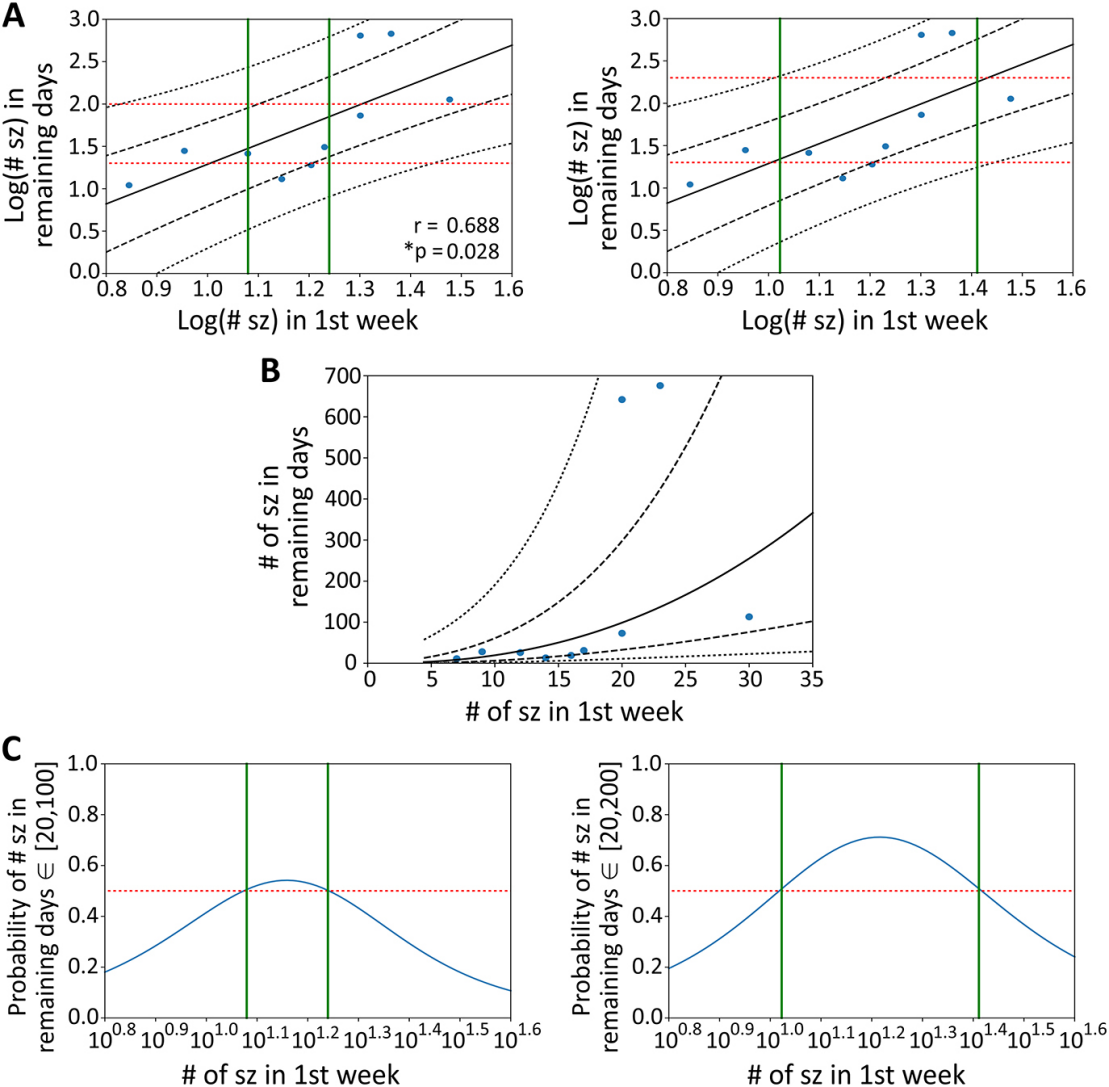
**Predictions on the number of seizures.** (A) The plot of Pearson correlation coefficients and Gaussian process modelling of the relation between the log number of seizures (sz) in the first week after onset of seizures and the number of seizures in the remaining recording days. Black lines represent the posterior Gaussian distribution (mean±one, and two s.d.). Red lines delimit a target interval of the number of seizures (left panel: from 20 to 100; right panel: from 20 to 200) in the remaining days and green lines delimit the interval for which there is more than 50% chance of observing such number of seizures (left panel: from 12 to 17; right panel: from 11 to 26) (*n*=10 animals). (B) Same as A, but with the axes exponentially transformed. (C) Probability that the number of seizures in the remaining days fall within the target interval of 20 to 100 (left panel) or 20 to 200 (right panel). Green lines delimit the interval above the 50% threshold mark (red line).

There was a significant and notable correlation between the number of seizures in the first week (from onset of seizures) and the number that occurred during the remaining recording period (day 8–day 30; Fig. 6A). We assessed this correlation for its predictive ability using a Gaussian process model with a linear kernel (Fig. 6A-C). This model of the data indicates, for example, that, if the number of seizures during the first week of seizures is between 12 and 17 (or between 11 and 26) for a given animal, that animal may be predicted to have between 20 and 100 (or between 20 and 200) more seizures in the following 23 days, with a confidence above 50% (Fig. 6C).

## DISCUSSION

In this study, we demonstrate that application of TeNT into the rat visual cortex produces a model of occipital cortical epilepsy. This chemically induced focal neocortical epilepsy model exhibits both clear ictal electrographic epileptic discharges and associated behavioural seizures that resemble human seizures. The high rate of induction of epilepsy associated with very low morbidity and mortality indicates that this is a well-tolerated and reproducible rat model of epilepsy, and may be considered a refinement of other models (such as the TeNT motor cortex model, where seizure activity can be accompanied by weight loss and death) (Wykes et al., 2012).

The major advantages of the TeNT model of focal epilepsy are that the epileptogenesis is not triggered by SE and does not rely upon neuronal loss or major disruption of tissue (Jiruska et al., 2010), and that it is a model of focal neocortical epilepsy that can be used to test focal treatments. TeNT is cleared from the brain a few days after local administration (Mellanby, 1989), but the disruption of VAMP persists, leading to recurrent spontaneous seizures over a long period (Mainardi et al., 2012; Ferecskó et al., 2015). This TeNT-induced occipital cortical epilepsy model does not start with SE and there is no significant cortical lesion. This is not atypical for many human neocortical epilepsies; indeed, in the largest surgical series to date, 8% of drug-resistant focal epilepsies that undergo resection have no detectable lesion at histology (Blumcke et al., 2017). Indeed, this is likely to underestimate the proportion of focal epilepsies with no histological lesion, as epilepsy surgery is naturally biased towards cases with an obvious target detected on MRI. Also, the progression of our model is predictable, as it has a relatively consistent latent period followed by progressively increasing seizure frequency and duration during the establishment of epilepsy, which reaches a plateau, and then resolves.

We defined repetitive epileptic discharges lasting for more than 10 s as ictal activity based on most seizures seen in humans (Jenssen et al., 2006; Hughes, 2009; Cook et al., 2016). This model exhibits both short (≤40 s) and long-lasting (>100 s) seizures with associated behavioural manifestations, and has markedly longer-duration (usually 90–140 s) seizures than those induced by TeNT injection into the motor cortex (which has mostly high-frequency bursts <1 s, or seizures lasting only few seconds) (Wykes et al., 2012). This is probably due to improved tolerability of higher doses of TeNT (15 ng) in the visual cortex, compared to the motor cortex where the dose was restricted to 12.5 ng in order to prevent mortality (Wykes et al., 2012). Although electrographic activity was recorded from a single electrode in the epileptogenic zone, seizure propagation and spread to other ipsilateral and/or contralateral brain regions could be observed from behavioural activity. These characteristics resemble the majority of human focal epilepsies. A limitation of this model is that, in some of the animals, the seizure frequency decreased between 4 and 5 weeks after the onset of spontaneous seizures. However, given the clinical need for new therapies and the paucity of focal neocortical models of chronic epilepsy, a model that recapitulates many features of human neocortical epilepsy is an invaluable tool for developing new treatments for focal neocortical epilepsy. Therefore, this model may be used to test for efficacy in developing new gene therapies and other novel treatment strategies.

A major advantage of using experimental models over human studies is that they are not confounded by treatment and other environmental factors can be controlled. Our data indicate that circadian cycles may affect seizure frequency, but not seizure duration. We found a trend towards higher seizure frequency in light periods, which is consistent with a lower seizure threshold in the sleeping state. The epileptogenic process may have an impact on the reciprocal influences between sleep-wake cycles and epilepsy (Quigg, 2000). Higher activity of seizures and interictal epileptiform discharges during non-rapid eye movement (NREM) sleep has been shown in some human epilepsies and in some experimental epilepsy models (Shouse et al., 2000; Hofstra and de Weerd, 2009). Approximately 20% of people with epilepsy have predominately nocturnal seizures (Schmitt, 2015). Thalamocortical synchronisation has been invoked to account for this association (Badawy et al., 2009; Timofeev et al., 2012). Circadian changes at many different levels have been proposed to explain the relationships between epilepsy and circadian rhythm (Quigg, 2000; Cho, 2012). Further studies to explore molecular changes driving the interactions of circadian rhythmicity, epileptogenesis and seizure occurrence will be important to understand the underlying mechanisms.

Clustering of seizures is often seen in human epilepsies (Karoly et al., 2016). In this model, seizures had a higher probability of occurring within a day of another seizure, and the overall seizure occurrence followed a periodic pattern in most of the animals. Seizure clustering could be due to positive-feedback mechanisms, whereas the negative dependency after clustering might be caused by negative-feedback mechanisms (Taubøll et al., 1991).

The total number of seizures experienced among individual animals in this model was highly variable. However, a positive correlation between the number of seizures in the first week and the remaining recording period permits a seizure prediction algorithm that can be applied to control for the variability of seizure frequency in animals enrolled in studies comparing treatments using this TeNT model of occipital cortical epilepsy. This reduction in variability has the potential to increase the power of comparisons of treatments, and consequently to reduce the number of animals needed.

In summary, an optimised rat model of occipital cortical epilepsy mimics focal neocortical epilepsy in humans, providing a non-SE- and non-lesion-induced epilepsy model for studying human epilepsy disorders and evaluating novel therapeutic strategies. We further provide a model for predicting the number of seizures. This can be used to split animals into treatment groups with similar expected seizure frequencies prior to treatment, reducing variability of the model and the number of animals needed for a given study.

## MATERIALS AND METHODS

### Animals

All animal experiments were conducted in accordance with the United Kingdom Animal (Scientific Procedures) Act 1986, and approved by the local ethics committee (University College London). A total of 21 male Sprague Dawley rats (6–12 weeks old, 260–330 g; Charles River, UK) were used (12 rats were injected with TeNT, 9 rats injected with normal saline as vehicle controls). Four Lister Hooded and three Long-Evans rats (8–12 weeks, 280–330 g) were used to validate the model in additional strains and for the intermittent photic stimulation. Animals were housed on a 12/12 h light/dark cycle (light cycle 7 a.m./7 p.m.), and maintained under controlled environmental conditions at ambient temperature 24–25°C and humidity 50–60% with environmental enrichment and *ad libitum* access to food and water. Animals were group housed and allowed to acclimatise to the new environment for at least 1 week before surgery, and were housed individually after surgery.

### Stereotactic surgery and implantation of wireless ECoG telemetry system

Rats were anaesthetised using isoflurane (2%) and head-fixed in a stereotaxic frame (Kopf, USA). A total of 15–15.6 ng of TeNT (gift of G. Schiavo, Cancer Research UK) in a final volume of 1 µl was injected into layer 5 of the right primary visual cortex at a rate of 200 nl/min (coordinates: 3 mm lateral and 7 mm posterior of bregma at a depth of 1 mm from pia). During the same surgery, an ECoG transmitter [A3028E-AA, Open Source Instruments (Chang et al., 2011), MA, USA] was implanted subcutaneously with a recording electrode wire positioned in the visual cortex above the site of TeNT injection and held in place by a screw. A reference electrode was placed in the contralateral frontoparietal cortex. Animals were single housed in Faraday cages and 24 h/7 days telemetric ECoG was continuously recorded immediately post-surgery, and carried on for up to 30 days counted from the onset of the first spontaneous electrographic seizure. For the rats in the vehicle control group, all the same surgical procedures were performed except 1 µl of 0.9% saline was injected in the right primary visual cortex instead of TeNT.

### Video-ECoG monitoring, ECoG data acquisition and analysis

IP cameras (M7D12POE or M7D12HD, Microseven) with an infrared video surveillance system time-locked to the ECoG were used for continuous 24 h/7 days telemetric video-ECoG monitoring. ECoG was recorded and processed using the Neuroarchiver tool (Open Source Instruments). The sampling rate of A3028E-AA implantable transmitters is 512 SPS (samples per second), which provides a frequency dynamic range (bandwidth) of 0.3–160 Hz and the voltage dynamic range is approximately 20 mV (−13 mV to +7 mV). Artefacts consisting of short-duration (<100 ms), high-amplitude ‘glitches’ were excluded. The ECoG was interpreted and epileptic seizures were detected manually by a neurologist analysing the entire ECoG dataset. The criteria used for detecting epileptic seizures were an evolution of frequency and amplitude over time with a sudden, repetitive, rhythmic, evolving and stereotypic abnormal electrographic activity with high amplitude (>2 times that of baseline) and a minimum duration of 10 s (Fisher et al., 2014; Pitkänen et al., 2005; Abend and Wusthoff, 2012; Shah et al., 2012).

### Behavioural seizure analysis and classification

To establish the classification of seizure behaviours corresponding to human seizure semiology, the top-down video footage time-locked to the ECoG trace was used to assess the behavioural correlates of electrographic seizures. A subset of seizures was randomly selected from animals with telemetric video-ECoG recordings for up to 5 weeks from TeNT injection. All seizures were verified with both video and ECoG. Seizure types were evaluated and classified by a neurologist according to the ILAE 2017 classification of seizure types (Fisher et al., 2017).

### Intermittent photic stimulation

To avoid any potential complications associated with poor eyesight of Sprague Dawley rats, intermittent flickering light stimulation was performed in awake Lister Hooded epileptic rats around 2 weeks after the onset of spontaneous seizures. To enhance the effect of photic stimulation, all other lights in the room were switched off during the test. An LED strip with high light intensity (>100 thousand foot-candles) attached to a board was wrapped around the cage. The frequency range of flickering light stimulation was from 1 Hz to 30 Hz. Different frequencies of photic stimulation were applied in the following sequence: 1 Hz, 3 Hz, 6 Hz, 9 Hz, 12 Hz, 15 Hz, 18 Hz, 21 Hz, 24 Hz, 27 Hz and 30 Hz, then followed by the reverse sequence of stimulation frequencies (from 30 Hz to 1 Hz). The duration of stimulation in each frequency was 20 s, with a rest period of 20 s before starting the next sequential frequency of stimulation.

### Statistical analysis

Data and statistical analyses were carried out as appropriate using Prism 6 (GraphPad) or Python (version 3.6, Anaconda, Inc., Texas, USA). To test whether the occurrence of seizures followed a Poisson process, the distributions of ISIs were compared to an exponential distribution with unknown mean using the Lilliefors adaptation to the Kolmogorov–Smirnov test (Lilliefors, 1969).

The autocorrelation was calculated to understand whether the seizures tend to arise in clusters. The peri-seizure histograms were computed by counting the intervals between all seizures and correcting for the fact that long intervals are less common due to the limited duration of the experiment of 30 days, i.e. each bin count was divided by 30 minus its central bin value in days. Two-tailed one-sample *t*-tests were used to test whether the mean of the histogram bins across rats (normalised by total seizure count) differed significantly from the counts expected from a uniform distribution.

The partial autocorrelation of daily seizure occurrence was computed to test the dynamical properties of seizure occurrence. Two-tailed one-sample *t*-tests were used to test whether the partial auto-correlation coefficients significantly differed from zero across rats.

A Gaussian process model using a linear kernel was fitted to the data in order to analyse the predictive power that the number of seizures in the first week of recording has upon the number of seizures occurring in the remaining days of the experiment.

## Supplementary Material

Supplementary information
