# Supplementary material for: Semiology, clustering, periodicity and natural history of seizures in an experimental occipital cortical epilepsy model
Source: Dis Model Mech. 2018 Dec 14;11(12):dmm036194. doi: 10.1242/dmm.036194 (PMC6307909; doi:10.1242/dmm.036194)
Supplement: Supplementary information [file dmm-11-036194-s1.pdf]

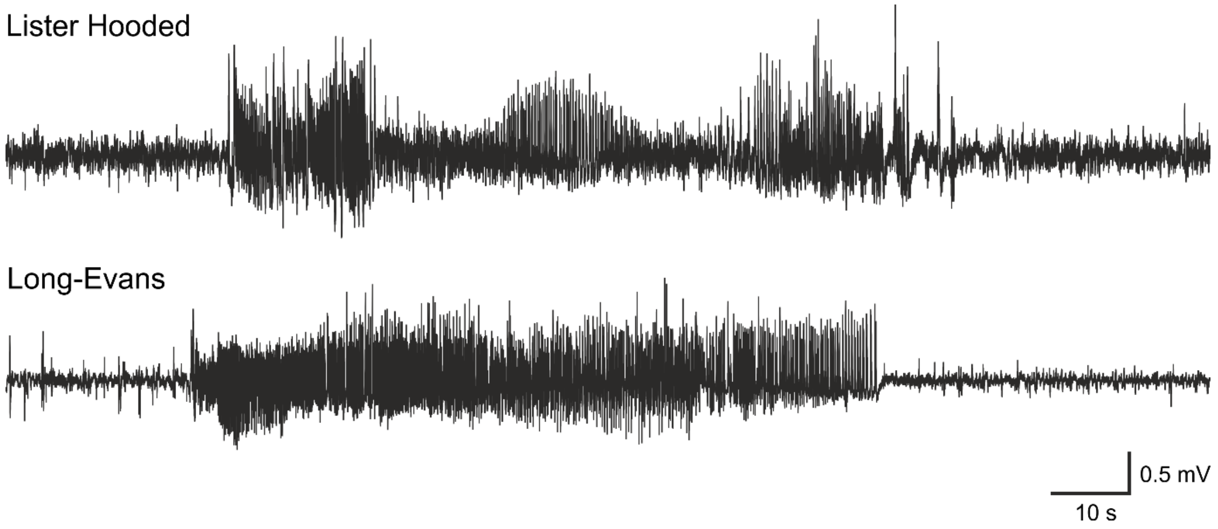

Figure S1. Representative seizures from Lister Hooded and Long-Evans rats. The TeNT model of occipital cortical epilepsy can be induced in other rat strains.

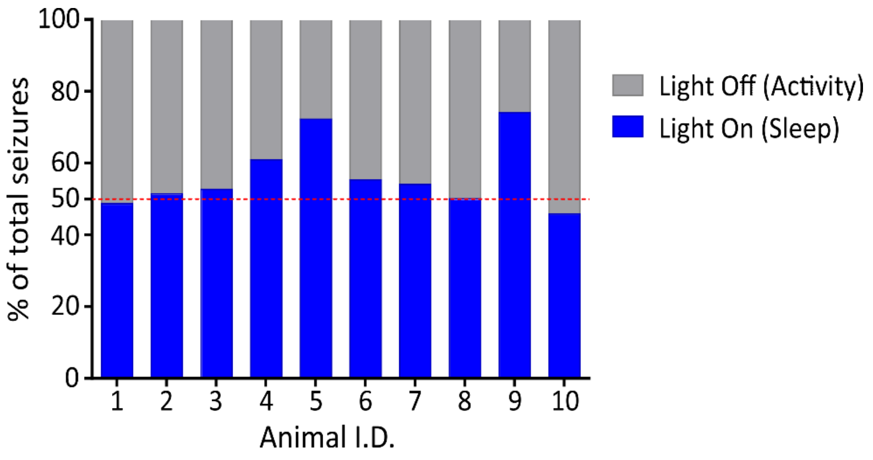

Figure S2. Distribution of seizure occurrence during day and night for individual animals. There are higher proportions of seizures during sleeping period in most of the animals.

Disease Models & Mechanisms • Supplementary information

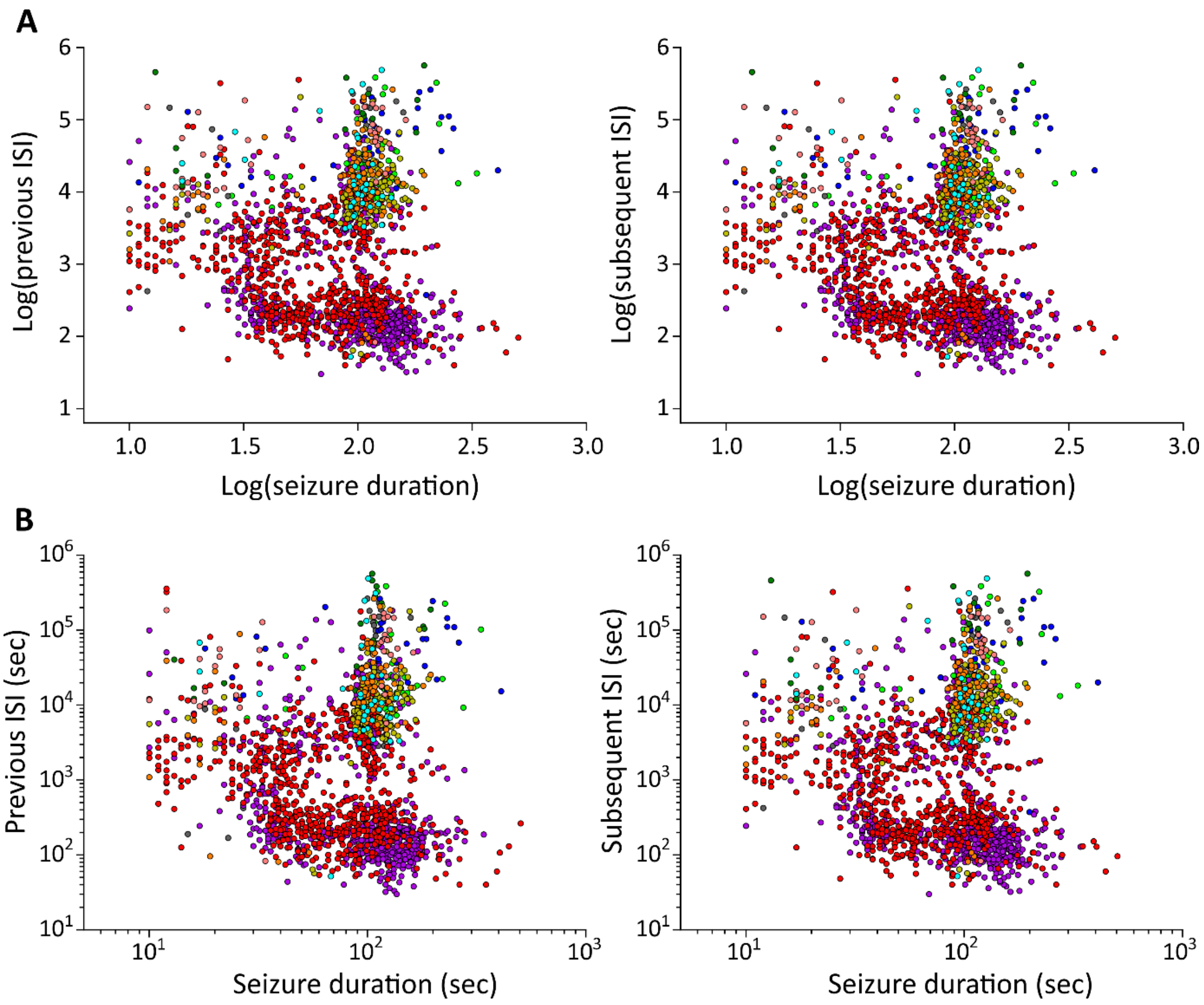

Figure S3. No obvious correlation between seizure duration and Inter Seizure Interval (ISI). Upper: Scatter plot of log of seizure duration with log of previous (left) and subsequent (right) ISI. Lower: Scatter plot of seizure duration with previous (left) and subsequent (right) ISI.

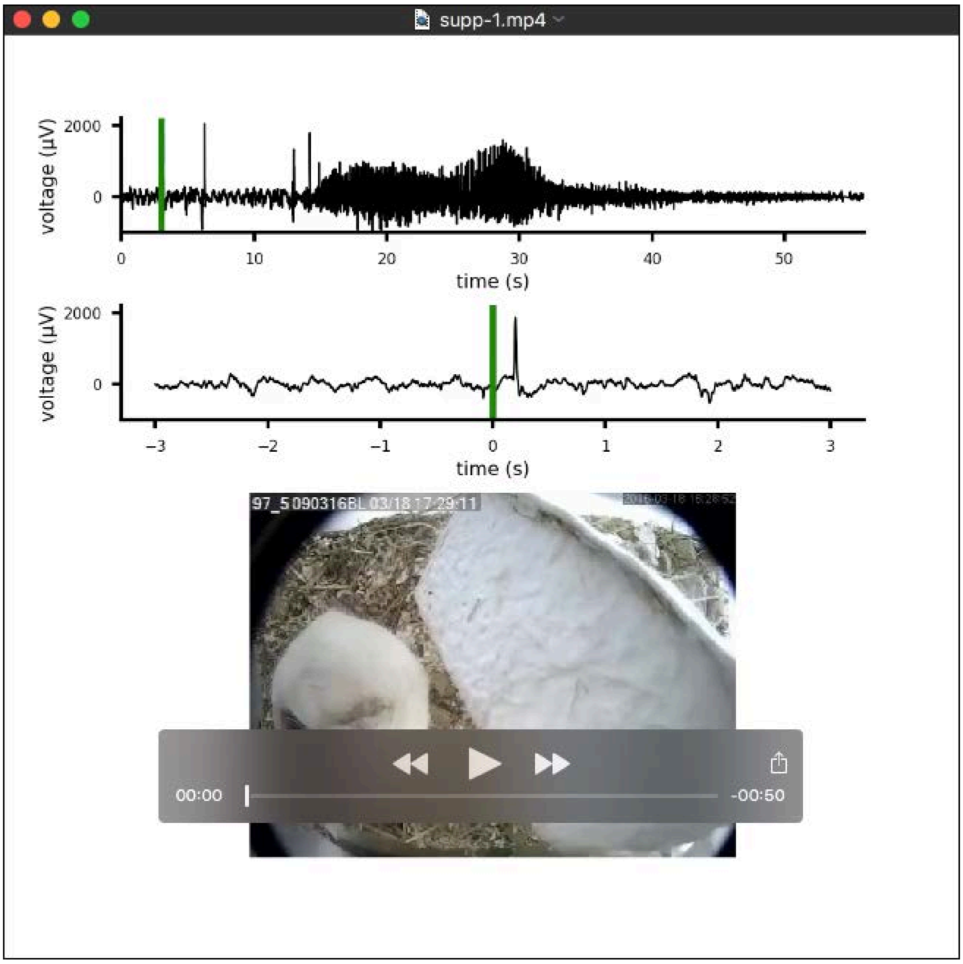

Movie 1. Non-motor seizure

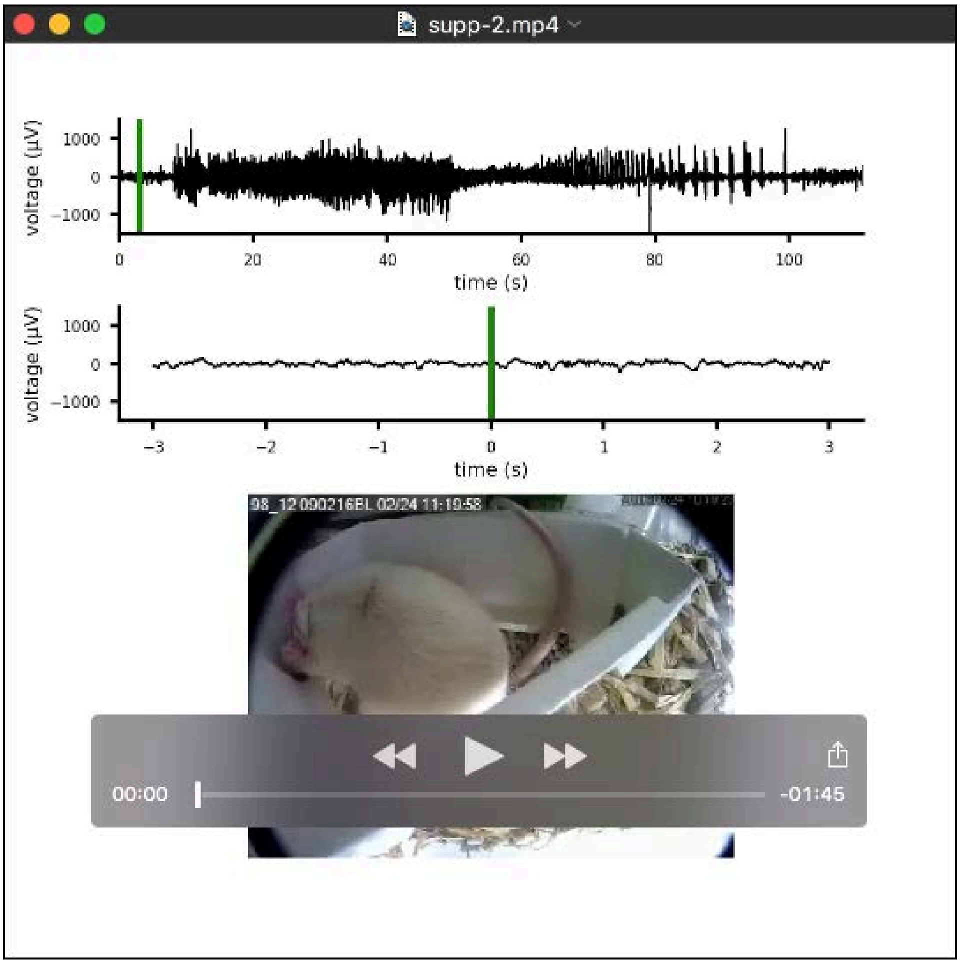

Movie 2. Focal seizure evolving to generalized tonic-clonic seizure
